# Longitudinal development of depression and anxiety during COVID-19 pandemic in Germany: Findings from a population-based probability sample survey

**DOI:** 10.3389/fpsyt.2022.1000722

**Published:** 2022-10-24

**Authors:** Katrin E. Giel, Peter Martus, Gregor Paul, Jan Steffen Jürgensen, Bernd Löwe, Lina Maria Serna Higuita, Annica F. Dörsam, Felicitas Stuber, Stefan Ehehalt, Stephan Zipfel, Florian Junne

**Affiliations:** ^1^Department for Psychosomatic Medicine and Psychotherapy, Medical University Hospital Tuebingen, Tuebingen, Germany; ^2^Institute for Medical Biometrics and Clinical Epidemiology, University Hospital Tuebingen, Tuebingen, Germany; ^3^Department of Gastroenterology, Hepatology, Pneumology and Infectious Diseases, Klinikum Stuttgart, Stuttgart, Germany; ^4^Department of Internal Medicine, Faculty of Medicine and University Hospital Cologne, University of Cologne, Cologne, Germany; ^5^Klinikum Stuttgart, Stuttgart, Germany; ^6^Department for Psychosomatic Medicine and Psychotherapy, University Hospital Hamburg Eppendorf, Hamburg, Germany; ^7^Public Health Authority, Stuttgart, Germany; ^8^Department for Psychosomatic Medicine and Psychotherapy, University Hospital Magdeburg, Magdeburg, Germany

**Keywords:** anxiety, COVID-19, depression, mental health, pandemic, population

## Abstract

The stress response to the COVID-19 pandemic might differ between early and later stages. Longitudinal data on the development of population mental health during COVID-19 pandemic is scarce. We have investigated mental health trajectories and predictors for change in a probability sample of the general population in Germany at the beginning and after 6 months of the pandemic. We conducted a longitudinal survey in a population-based probability sample of German adults. The current study analyzed data from a first assessment in May 2020 (T1; *N* = 1,412) and a second in November 2020 (T2; *N* = 743). Mental health was assessed in terms of anxiety and depression using the Patient Health Questionnaire-4 (PHQ-4). Mental health outcomes at T1 were compared with PHQ-4 norm data. Trajectories over time were investigated based on outcome classifications of PHQ-4 scores. Predictors of mental health outcomes and change were identified using multiple regression analysis. In spring 2020, participants showed significantly higher PHQ-4 scores as compared to the norm data, however, overall anxiety and depression remained low also 6 months later. 6.6% of respondents showed a mental health deterioration in autumn 2020, entering subclinical and clinical ranges, outweighing the proportion of people with improved outcomes. Sociodemographic variables associated with mental distress at T1 were mainly not predictive for change at T2. Even under prolonged pandemic-related stress, mental health remained mainly stable in the general population. Further development of the considerable subgroup experiencing deterioration of depression and anxiety should be monitored, in order to tailor prevention and intervention efforts.

## Introduction

From a mental health perspective, the COVID-19 pandemic can be understood as a global stress induction. Large population groups live under recurrent lockdown situations and threat of a potential infection, experiencing a deprivation of resources and rewarding experiences while mostly having limited control and perspective regarding the situation. The course of the pandemic induces different stages and levels of stress which match well with seminal stress models (1, 2): While the first lockdown in spring 2020 might have induced acute stress, the ongoing pandemic might qualify as a chronic stressor. Hence, the pandemic provides us with novel insights into how individuals cope with stress and about who stays healthy and who is specifically vulnerable to adverse outcomes of chronic stress, including the development of mental symptoms and disorders. This knowledge is pivotal to inform government and health care decisions targeting mental health sequelae of the pandemic (3, 4). However, major methodological limitations of the evidence have been criticized, including a wide reliance on convenience samples (5–8) and a lack of longitudinal data (7, 9, 10). Two large representative surveys from the US (11) and UK (12) investigating pre-post-pandemic mental health outcomes found increased distress in the general population early after the COVID-19 outbreak. The few representative longitudinal studies draw a more differential picture: Data comparing multiple assessments during early stages of the pandemic indicate no changes in mental health outcomes (10, 13, 14), or even a decrease in depression and anxiety over the first 20 weeks of lockdown (15). The few representative studies analyzing individual mental health trajectories identify most people as resilient, while 7% to 11% of individuals reported mental health decline (9, 10, 16) vs. 9–12% experiencing improvements (10, 16). This pattern in mental health development over time has also been found in population-based surveys conducted in Germany (17, 18): Based on the same instrument as used in the present study to assess anxiety and depression, an initial increase in anxiety and depression was found in early stages of the pandemic, which was again reduced during the second wave of the pandemic (18), but overall higher scores of anxiety and depression were reported peri-pandemic as compared to pre-pandemic years (17). Consistently, a recent meta-analysis on lockdown effects on population mental health concludes that most individuals stay mentally healthy (8). Importantly, most of these data stem from the initial stage of the pandemic (8–15), a stage of adaption to an acute stressor (1) as well as stepwise withdrawal of lockdown measures (10). However, mental health might be affected differently along the different stages of the pandemic.

We contribute to the evidence on population mental health during the COVID-19 pandemic by presenting longitudinal data from two assessments within a period of 6 months from a probability sample survey in a German metropole region. We used the Patient Health Questionnaire-4 (PHQ-4) (19, 20) as validated self-report instrument to assess symptoms of depression and anxiety in spring 2020 (T1) and in autumn 2020 (T2). The second assessment point was chosen as in autumn 2020, this was the beginning of the second infection wave and also the second lockdown in Germany, and we hypothesized that these circumstances might impact population mental health. At T1, the 7-day incidence of COVID-19 infections was 5.7 / 100.000 inhabitants in Germany and 7.5 in Stuttgart; at T2, the 7-day incidence was 153.1 in Germany and 137.6 in Stuttgart.

We hypothesized that on average, we will find increased levels of anxiety and depression (a) at T1 as compared to representative norm data, and (b) at T2 as compared to T1 due to reapplied lockdown measures. We expected (c) a majority of the sample to be resilient to mental distress and a small group to show trajectories of impaired mental health and (d) that we will be able to identify sociodemographic predictors for increased distress at T1 and the change between T1 and T2. We tested female gender, younger age, lower education background, living alone and living with children as they have been previously identified as predictors for mental distress early in the pandemic (11, 12, 15, 18). Additionally, we looked at Body Mass Index (BMI) as exploratory variable as elevated BMI has been found to be associated with higher levels of anxiety and depression (21) and as BMI is a proxy of eating behavior which, in some individuals, can serve as an emotion regulation strategy under stressful conditions (22).

## Methods

The present study is reported according to the STROBE statement (23).

### Study design and recruitment

This survey is a subproject of a longitudinal serological investigation of undetected SARS-CoV-2 infection in the general population. Data was derived from a probability sample of the adult general population living in Stuttgart, Germany. Major confinement measures throughout the pandemic, including lockdowns, were in-place on a nationwide level in Germany, hence the situation of the population of Stuttgart is comparable with circumstances in other parts of the country.

### Measures

Mental health was assessed in terms of core symptoms of anxiety and depression using the PHQ-4 (19, 20) which is a widely used screening tool comprised of two items assessing anxiety (GAD-2) and two items assessing depressive symptoms (PHQ-2). The PHQ-2 comprises the DSM-IV core criteria for depressive disorders which are assessed for the last 2 weeks (20), while the GAD-2 assesses the two core criteria for generalized anxiety disorder (20), which have been found to be also good screening approaches for panic, social anxiety and post-traumatic stress disorder (24). The PHQ-4 total score, a sum of PHQ-2 and GAD-2 scores, ranges from 0 to 12 with scores ≥ 6 ≤ 8 considered as yellow flag and scores ≥ 9 considered as red flag for the presence of anxiety and depression (20). The PHQ-4 is a very widely used brief screening tool for anxiety and depression with excellent psychometric qualities (20). We additionally assessed sociodemographic variables.

### Procedure

Adult members of 4,400 households in Stuttgart were invited *via* postal letters to participate in the study. This initial sample was drawn based on data from the residents' registration office and was representative for the adult population living in Stuttgart. Only one single person was invited per household. The first assessment point took place in the second week of May 2020, which was toward the end of the first pandemic wave in Germany. Study participants were re-invited in the last week of November 2020, which was at the beginning of the second lockdown in Germany. Participants were offered to fill in either a paper or an online version of the survey with identical content. No further exclusion criteria applied.

### Ethics statement

All procedures contributing to this work comply with the ethical standards of the relevant national and institutional committees on human experimentation and with the Helsinki Declaration of 1975, as revised in 2008. All procedures involving human subjects were approved by the ethics committee of the Medical Faculty Tuebingen and the University Hospital Tuebingen (271/2020BO1). Written informed consent was obtained from all subjects.

### Statistical analyses

Primary aim of the study was to investigate mental health trajectories assessed by the PHQ-4 in the general population at the beginning of the pandemic and after 6 months. Predictors for both, baseline and change after 6 months should be identified. For comparison, we used raw data from the PHQ-4 validation study (20). To address responder bias, relevant characteristics at baseline were compared between responders and non-responders using chi-squared test (full df or one df in case of ordinal variables) and *t*-tests (normally distributed data) or Mann-Whitney tests (non-normally distributed data). Normality was assessed by inspection of skewness and kurtosis (both had to be between −1 and +1).

PHQ-4 was analyzed quantitatively and according to a classification proposed by Löwe et al. (20) (see above). Like previously applied by other workgroups (10), we had a specific focus on individual trajectories between T1 and T2 and classified the study sample into participants who remained stable within the respective PHQ-4 band (below 6, ≥ 6 ≤ 8 and below 8), those who improved as they were moving to a lower band and those who deteriorated as they were moving to a higher band.

Change of PHQ-4 was assessed by *t*-tests for paired samples (continuous scale), and by sign tests (categorical scale). Associations between quantitative predictors and PHQ-4 at baseline were assessed by linear models (Pearson correlations, ANOVA, including Tukeys B for pairwise comparisons, Curve fit for inspection of quadratic terms, and multiple regression analysis). The same methods were used to assess associations with change of PHQ-4 scores. No imputation was performed and change over time was analyzed only for subjects who participated at T2. This was an exploratory study, thus the chosen level of significance (0.05 two-sided) is not strictly confirmatory and not adjusted for multiple testing. The analyses were carried out using SPSS release 26 (Armonk, NY: IBM Corp). For the Sankey plots, the package R (Vienna, Austria: R Foundation for Statistical Computing) was used.

## Results

### Sample characteristics

The baseline sample at T1 comprised 1,412 participants (32.1% response rate) with a mean age of 50.7 ± 18.7 years of which 48.1% were females. 18.3% were living alone, 21.3 % were living with one or more children (Table 1). 64.5 % were employed, and of those working, 51.6% were predominantly and 27% were completely working from home.

**Table 1:** Sociodemographic variables in survey responders vs. non-responders at follow-up.

**Variable**	**Non-** **responders in** **follow-up**	***n***	**Responders in** **follow-up**	***n***	***P*** **-value** **responder bias**	**Responders in** **follow-up**	***n***	***P*** **-value** **follow-up vs.** **baseline**
Age (yrs); M ± SD	50.7 ± 18.7	667	45.3 ± 15.8	741	<0.001^MW^	N.A.		N.A.
**Sex;** ***n*** **(%)**		669		743	0.008^Chi^	N.A.		N.A.
Male	347 (51.9%)		333 (44.8%)					
Female	322 (48.1%)		410 (55.2%)					
Missing values	0		0					
BMI (kg/m^2^); M ± SD	25.5 (± 4.6)	659	24.9 (± 4.7)	733	0.001^MW^	24.8 (± 4.6)	732	0.48^WT^
**Education;** ***n*** (%)		622		711	0.001^LL^	N.A.		N.A.
None	79 (12.7%)		40 (5.6%)					
Vocational training	220 (35.4%)		232 (32.6%)					
Bachelor degree	63 (10.1%)		91 (12.8%)					
Master degree	120 (19.3%)		177 (24.9%)					
Diploma	140 (22.5%)		171 (24.1%)					
Missing value	47		32					
**Persons in household;** ***n*** (%)		667		741	0.36^LL^	N.A.		N.A.
1	122 (18.3%)		128 (17.3%)					
2	314 (47.1%)		343 (46.3%)					
3	116 (17.4%)		118 (15.9%)					
4	79 (11.8%)		118 (15.9%)					
5 or more	36 (5.4%)		34 (4.6%)					
Missing values	2		2					
**Children in household;** ***n*** (%)		668		742	0.14^LL^	N.A.		N.A.
0	526 (78.7%)		563 (75.9%)					
1	74 (11.1%)		81 (10.9%)					
2	52 (7.8%)		80 (10.8%)					
3 or more	16 (2.4%)		18 (2.4%)					
Missing value	1		1					

At T2, 743 people (52.8%) participated in the survey. Responders were significantly younger, more often female and reported a lower BMI at baseline. None of the remaining characteristics were different between responders and non-responders (Table 1).

### Mental health outcomes at baseline and their predictors

**Figure 1:**
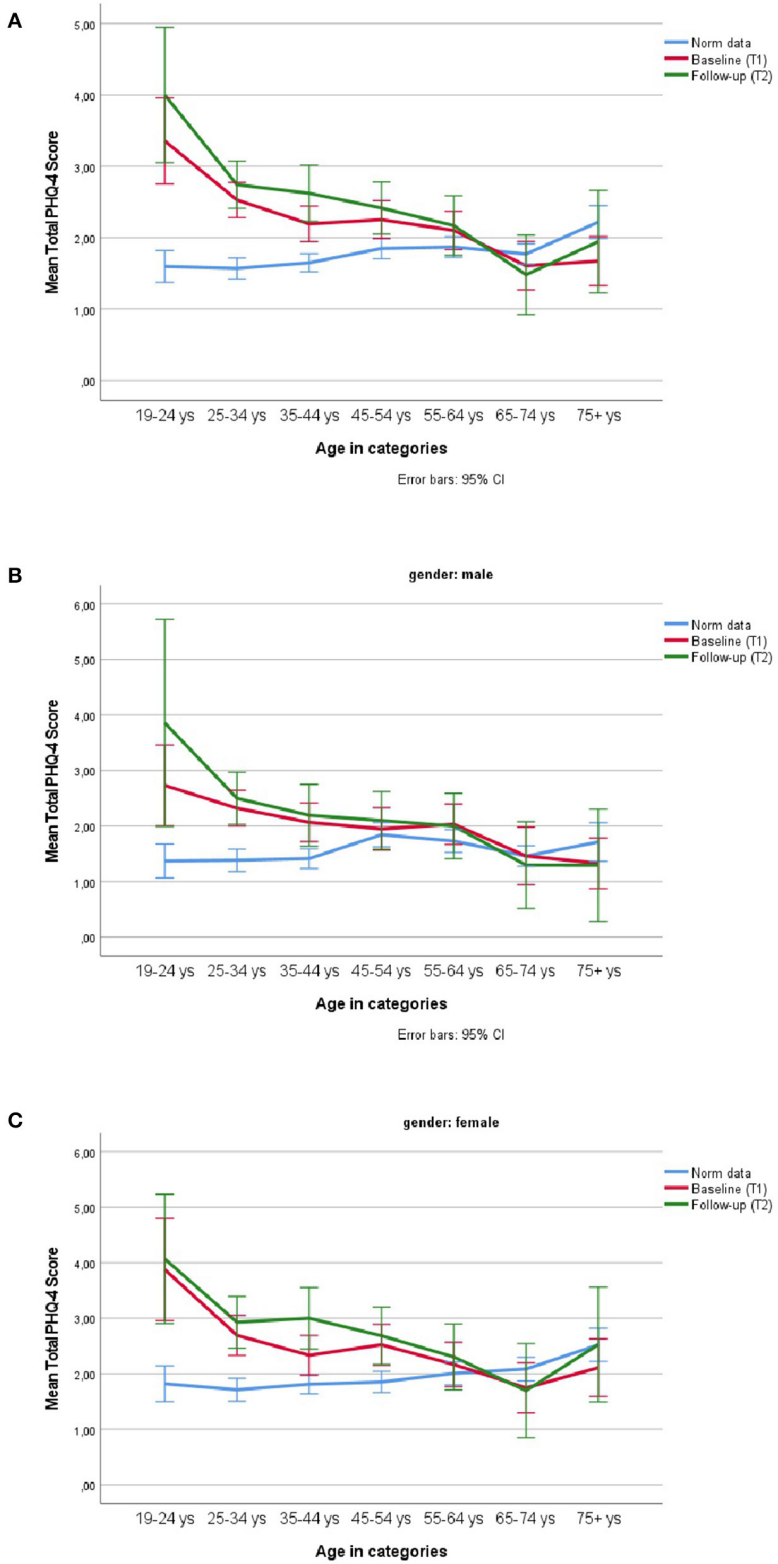
PHQ-4 mean scores at T1 and T2 in the survey population as compared to PHQ-4 normative data in different age groups in **(A)** the total sample, **(B)** in males, and **(C)** in females.

PHQ-4 scores at T1 were significantly higher in our sample as compared to the norm data (see Figure 1).

Higher PHQ-4 scores where observed for younger age (*r* = −0.158, *P* < 0.001), especially for participants between 19 and 24 years (Anova: *F*_(6,1394)_ = 8.41, *η*^2^ = 0.036, Tukeys B *P* < 0.01,). Females were more affected than males [*t*_(1,399)_ = −3.98, Cohen's d = 0.21, *P* < 0.001]. Figure 1 shows age and gender effects compared to PHQ-4 norm values in the German population. Participants with underweight (BMI < 18.5 kg/m^2^) and obesity (BMI > 30 kg/m^2^) were more affected than those with BMI between 18.5 and 30 kg/m^2^ [quadratic term, b = 0.104, *t*_(1,382)_= 3 .85, *P* < 0.001]. People with academic education were less affected than the remaining groups [b = −0.182, *T*_(1,326)_ = −4.12, *P* < 0.001]. There was no effect for the number of children [b = 0.148, *t*_(1,401)_ = 1.90, *P* = 0.058] and an unclear pattern for household size [ANOVA *F*_(4,1,396)_ = 3.94, *η*^2^ = 0.01, *P* = 0.003, linear trend *P* = 0.093, quadratic *P* = 0.048]. In a multiple regression analysis, all predictors [*r*^2^ adjusted = 0.053, age, b = −0.022, *t*_(1,300)_ =−6.17, *p* < 0.001; gender, b = 0.36, *t*_(1,300)_ = 2.96, *p* = 0.003; BMI linear, b = 0.133, *t*_(1,300)_ = 1.69, *p* = 0.092; BMI quadratic, b = 0.066, *t*_(1,300)_ = 2.40, *P* = 0.017; education, b = −0.133, *t*_(1,300)_ = −3.00, *P* = 0.003] were significant. Results were similar for the subscale PHQ-2 and less pronounced for the subscale GAD-2 (Supplementary material 1).

### Longitudinal mental health trajectories

**Figure 2:**
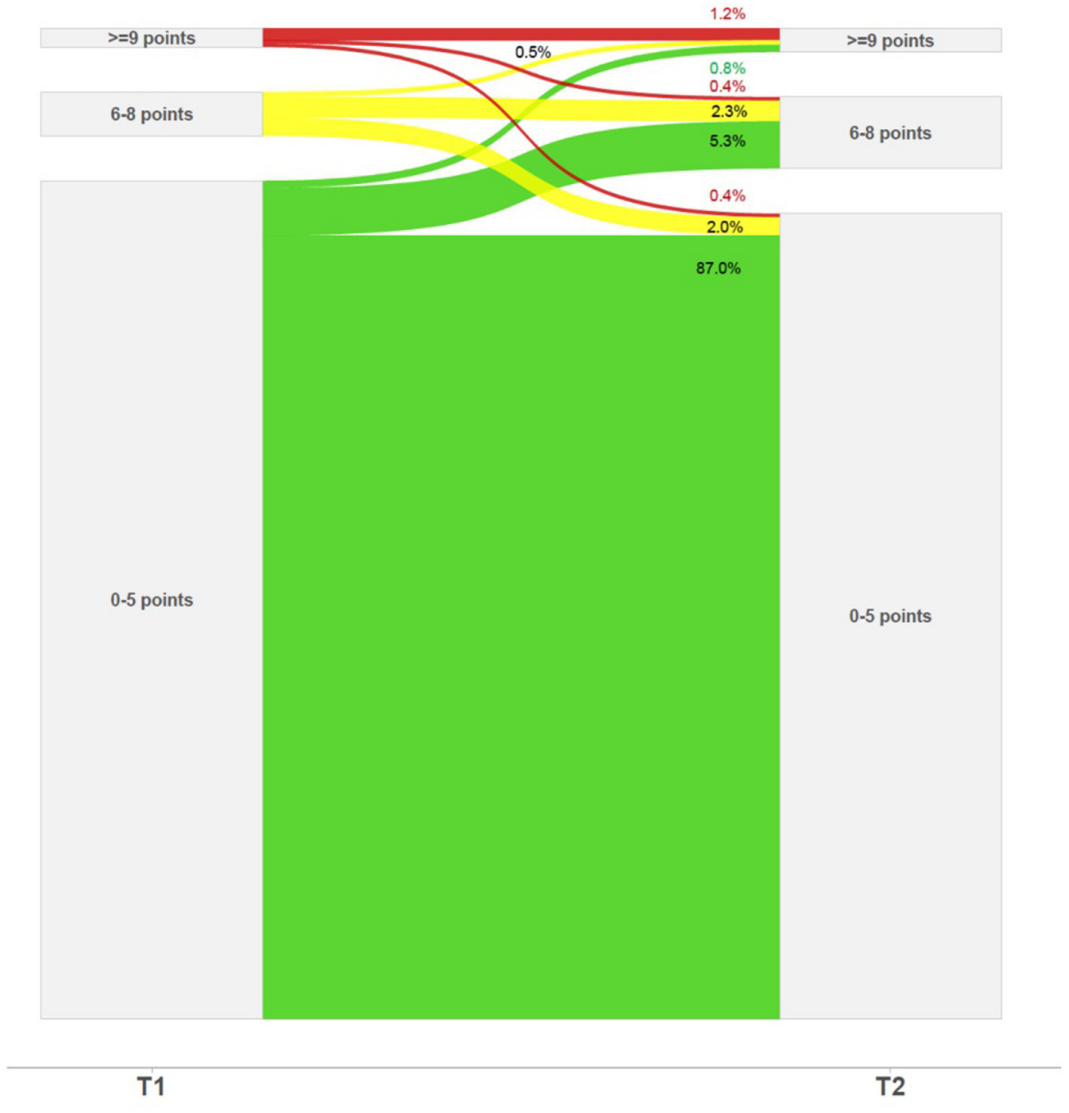
PHQ-4 mental health trajectories between T1 and T2.

In the quantitative analysis, changes of the PHQ-4 score and the PHQ-2 subscale score were highly significant [cohen's d = 0.16 total, (subscale 0.18), (*t*_(740)_ = 4.24, (4.99), *P* < 0.001 each] whereas the change in the GAD-2 subscale was less pronounced [cohen's d = 0.08, *t*_(740)_ = 2.13, *P* = 0.03] (Table 2). Figure 2 shows that a vast majority of participants (87%) had stable PHQ-4 scores within the good mental health range. Significantly more participants (6.6%, *n* = 49) showed a deterioration of mental health at T2, as compared to those showing a mental health improvement (*n* = 49 vs. *n* = 21, 2.8%, *P* = 0.001, exact binomial test). Most of the deteriorations indicated migrating from good health into the “yellow flag” range and a small proportion moving in the “red flag” range.

**Table 2:** Mental health outcomes in survey responders vs. non-responders at follow-up.

**Variable**	**Non-** **responders in** **follow-up**	***N***	**Responders in** **follow-up**	***n***	***P*** **-value** **responder bias**	**Responders in** **follow-up**	***n***	***P*** **-value** **follow-up vs.** **baseline**
PHQ-4 sum score; M ± SD	2.2 ± 2.2	741	2.3 ± 2.2	660	0.94^MW^	2.5 ± 2.4	741	<0.001^WT^
**PHQ-4 categorized;** ***n*** (%)		741		660				
Good mental health	690 (92.6%)		608 (92.1%)		0.67^LL^	663 (89.5%)		0.001^ST^
Yellow flag	36 (5.4%)		40 (6.1%)			59 (8.0%)		
Red flag	15 (1.9%)		12 (1.8%)			19 (2.6%)		
PHQ-2 sum score; M ± SD	1.2 (±1.1)	742	1.2 (±1.2)	663	0.95^MW^	1.4 (±1.3)	741	<0.001^WT^
**PHQ-2 categorized;** ***n*** (%)					0.46^LL^			<0.001^ST^
Good mental health	671 (90.4%)		592 (89.3%)			634 (85.6%)		
Yellow flag	58 (7.8%)		57 (8.6%)			85 (11.5%)		
Red flag	13 (1.8%)		14 (2.1%)			22 (3.0%)		
GAD-2 sum score; M ± SD	1.0 (±1.2)	741	1.1 (±1.3)	662	0.88^MW^	1.1 (±1.3)	741	0.03^WT^
**GAD-2 categorized;** ***n*** (%)					0.98^LL^			0.057^ST^
Good mental health	668 (90.1%)		597 (90.2%)			649 (87.6%)		
Yellow flag	56 (7.6%)		50 (7.6%)			74 (10.0%)		
Red flag	17 (2.3%)		15 (2.3%)			18 (2.4%)		

BMI, Body Mass Index; Chi, Chi square; GAD-2, Subscale assessing depression of the Patient Health Questionnaire-4; LL, linear-by-linear association test; M, mean; MW, Mann–Whitney-U test; PHQ-2, Subscale assessing depression of the Patient Health Questionnaire-4; PHQ-4, Patient Health Questionnaire-4; SD, standard deviation; ST, Sign test; WT, Wilcoxon test.

PHQ categories: Good mental health = sum scores below six for the PHQ-4 and below three for the subscales; Yellow flag = sum scores between six and eight for the PHQ-4 and sum scores of three or four for the subscales; Red flag = sum scores of nine or larger for the PHQ-4 and sum scores of five or larger for the subscales.

### Predictors of mental health changes after 6 months

In contrast to the cross-sectional baseline analysis, except for BMI, none of the predictors investigated were significantly associated with the change in PHQ-4 scores (continuous scale) during the observation period. There was a small significant effect (*r* =-0.087, *P* < 0.02) that participants with a higher BMI showed less deterioration as compared to people with lower BMI.

## Discussion

The present longitudinal survey assessed depression and anxiety trajectories over 6 months of the COVID-19 pandemic in a large German population-based probability sample.

We replicated findings showing mental health impairments early in the pandemic (11, 12, 18), with females (18, 25), younger people (18) and people with lower education level being more affected (7). Moreover, we found people on both poles of the BMI spectrum to be more affected, while underweight/obesity might be associated with higher vulnerability toward stress and generally increased mental health burden (21). The BMI-related effects in our sample might partly also mirror current longitudinal trends indicating an increased incidence of eating disorder diagnoses over the first months of the pandemic (26). Regarding potential sex differences in mental health outcomes, it is important to consider several aspects: First of all, longitudinal representative trajectory data on mental health does not report sex differences (10, 15, 16), highlighting again the importance to differentiate between initial and ongoing reaction to the crisis. Secondly, sampling effects could influence data as especially in convenience samples, a significant larger group of participants is female (7). Third, population-based surveys are usually brief and cover the most common mental health outcomes, and while women might just be more likely to endorse symptoms of anxiety and depression, surveys potentially neglect symptoms that are more common experienced in males under stressful conditions (27). Finally, elevated rates of anxiety and depression in females early in the pandemic might partly reflect common gender roles rather than biological sex differences, for instance, women juggling employment and care work under lockdown conditions (27).

Our hypothesis of overall longitudinal deterioration in anxiety and depression 6 months later was supported. Yet, most people remained stable in the range of good mental health, and these individual trajectories support recent evaluations that the mental health of most participants remains stable despite pandemic-induced stress (8–10, 16). The trajectory data also matches with our theoretical argument related to assumptions of general stress models (1, 2): Initial increased mental health burden might mirror acute stress in the general population during the first lockdown in spring 2020. Over half a year, the majority of the population shows resilience toward the ongoing pandemic, however there is also a substantial group showing metal health deterioration under this now chronic stress situation. development of mental symptoms and disorders.

In contrast to trajectory data from UK covering earlier time intervals (10, 16), the group in our sample experiencing mental health deterioration was slightly smaller, still, there were clearly more people declining than improving in mental health, while these contrasting groups were nearly equal in the UK surveys (10, 16). Our data covers a comparably longer time interval, re-assessing the sample after reapplication of nationwide lockdown measures in Germany, and this might explain why we found less improvement regarding anxiety and depression. BMI was the only variable predicting mental health change over 6 months, though this effect was small and should be interpreted with caution. However, the evidence on who is vulnerable in the long run of the pandemic is still limited, and also a recent study investigating mental health trajectories concludes that most of the predictors for distress in early pandemic stages were less consistently associated with longitudinal mental health trajectories (10). There is preliminary evidence for pre-existing illness, socioeconomic status and ethnicity to predict long-term mental health deterioration during COVID-19 pandemic (16).

Germany is a high-income country, and, in light of this, it is important to consider that trajectories in population mental health may also be related to the national health and social care systems, as well as specific government responses to the crisis and available resources in the society. Indeed, Germany has taking several measures in order to mitigate the impact of the pandemic on people's live circumstance, for instance, financial reimbursement was widely implemented in Germany for individuals unable to work during lockdowns. In contrast, economic uncertainty throughout the pandemic might be more severe and might impact more strongly mental health outcomes in developing countries (28).

### Strengths and limitations

In the present study, we report data on longitudinal mental health outcomes during COVID-19 pandemic from a population-based probability sample. As such, it overcomes some of the methodological weaknesses of online survey data (5) which currently forms most of the evidence based on mental health outcomes during the pandemic (7). Our survey participants were invited *via* mail to their postal address, which allows also people to participate who would have been digitally excluded. Our data covers an interval of 6 months, and we rely on a widely used instrument assessing anxiety and depression (20). The PHQ-4 is a brief screening instrument with excellent psychometric qualities (20), allowing for an ecological assessment of mental health outcomes, which is an advantage especially in large surveys. However, at the same time, we did not cover other aspects of mental health, for instance such as insomnia. Further limitations comprise that the study protocol was not pre-registered, we cannot compare to pre-pandemic data; our sample exclusively stems from an urban background, and the survey lacks information about variables which have previously been identified to influence mental health outcomes, such as ethnicity and income (15, 16), sense of coherence (29) or media use (30). The PHQ-4 norm data was published in 2010 which dates back several years from the implementation of the present study. In the course of time, the prevalence of anxious and depressive symptoms might have varied due to factors unrelated to the pandemic. We found a responder bias between T1 and T2 assessment, however, none of the respective variables was strongly associated with mental health change over time. It should be noted that at T1, the concept of predictors is weaker than in the longitudinal setting at T2.

### Perspectives and future studies

Future research efforts are needed for an in-depth investigation of long-term trajectories of mental health throughout the pandemic and also post-pandemic (7). For instance, it will be insightful to analyze the development through winter and spring 2020/21 prolonged lockdown conditions in many countries, but also throughout winter 2022 which was characterized by altered strains and circumstances with a novel virus variant. Taking a longer-term perspective, it will be an important question if elevated mental health burden throughout the pandemic puts individuals at risk to develop clinical mental health conditions, and, on a population-level, if and when overall mental health status recovers to pre-pandemic levels. A further pivotal line of research focuses on predictors of both, mental health deterioration and mental resilience throughout the pandemic on a population level and in vulnerable subgroups (17, 31). Knowledge on such risk and protective factors will inform tailored prevention efforts and intervention strategies for future pandemic circumstances. Beyond, and taking a more global perspective, a stronger differentiation of how population mental health has been affected in countries with different government measures, socio-economic levels and health care systems is necessary in order to better understand which political and administrative interventions might be harmful and helpful.

### Conclusions

Our longitudinal population-based study contributes to the literature on mental health outcomes during COVID-19 pandemic by reporting trajectory data beyond questionnaire mean scores. These data show that most individuals remain in a stable and healthy range regarding symptoms of anxiety and depression under prolonged pandemic-related stress. Our study indicates that vulnerability factors differ over the course of the pandemic: While most of those initially vulnerable to acute stress might quickly adapt (15), other groups vulnerable to long-term effects of stress evolve over time.

Importantly, a considerable subsample did experience a deterioration of depression and anxiety symptoms over 6 months. Research efforts on long-term peri- and post-pandemic trajectories of mental health are needed in order to tailor prevention efforts for future pandemic circumstances (4, 32) and to offer support to vulnerable individuals (4), including adapted dissemination strategies, digital and low-threshold interventions (33).

## Data availability statement

Raw data related to the present study will be made available by the corresponding author upon reasonable request.

## Ethics statement

The studies involving human participants were reviewed and approved by Ethics Committee at the Medical Faculty of the University Tübingen and the University Hospital Tübingen. The patients/participants provided their written informed consent to participate in this study.

## Author contributions

KEG, GP, JSJ, SE, and FJ designed the study and implemented core study procedures. AD and FS contributed to preparation of the survey and supported survey conduction and data handling. PM is the responsible biostatistician. PM and LMSH conducted the data analysis and prepared the figures. BL provided data for parts of the data analysis. PM, KEG, SZ, and FJ interpreted the data. KEG drafted the manuscript. All authors critically revised it and approved the final manuscript.

## Conflict of interest

The authors declare that the research was conducted in the absence of any commercial or financial relationships that could be construed as a potential conflict of interest.

## Publisher's note

All claims expressed in this article are solely those of the authors and do not necessarily represent those of their affiliated organizations, or those of the publisher, the editors and the reviewers. Any product that may be evaluated in this article, or claim that may be made by its manufacturer, is not guaranteed or endorsed by the publisher.
