# Supplementary material for: Longitudinal development of depression and anxiety during COVID-19 pandemic in Germany: Findings from a population-based probability sample survey
Source: Front Psychiatry. 2022 Oct 24;13:1000722. doi: 10.3389/fpsyt.2022.1000722 (PMC9637933; doi:10.3389/fpsyt.2022.1000722)
Supplement: Supplementary file 1 [file Data_Sheet_1.docx]

**Supplementary Material 1**

## Mental Health Outcomes at Baseline and their Predictors differentiated by symptom category

1. **Depression (PHQ-2)**

Higher PHQ-2 scores where observed for younger age (r=-0.19, *P*<0.001), especially for participants between 19 and 24 years (Anova: F_6,1398_ =11.367, Tukeys B *P*<0.05). Females were more affected than males (t_1407_=-3.319, *P*=0.03). Participants with underweight (BMI < 18.5 kg/m²) and obesity (BMI > 30 kg/m²) were more affected than those with BMI between 18.5 and 30 kg/m² (quadratic term, b=-0.104, F_1386_=11.040, *P*<0.001). Overall, people with different academic education significantly differed in PHQ-2 scores (Anova: F_4,1326_ =6.456, *P*<0.001), however post-hoc tests revealed also some overlap between educational backgrounds, with a tendency for people with higher education being less affected (Tukeys B *P*<0.05). There was no effect for the number of children (ANOVA F_3_,_1403_=2.531, *P*= 0.056) on PHQ-2 score and again an unclear pattern for household size.

In a multiple regression analysis, all predictors were significant for PHQ-2 score (r^2^ adjusted = 0.061, age, b=-0.204, t_1308_=-7.351, p<0.001; gender, b=0.068, t_1300_=2.463, p = 0.14; BMI quadratic, b=0.098, t_1300_=2.906, *P* = 0.004; education, b=-0.062, t_1300_=-2.263, *P* = 0.024).

1. **Anxiety (GAD-2)**

Higher GAD-2 scores where observed for younger age (r=-0.95, *P*<0.001), especially for participants between 19 and 24 years (Anova: F_6,1396_ =3.778, Tukeys B *P*<0.05). Females were more affected than males (t_1405_=-3.755, *P*=0.045). Participants with underweight (BMI < 18.5 kg/m²) and obesity (BMI > 30 kg/m²) were more affected than those with BMI between 18.5 and 30 kg/m² (quadratic term, b=--1.01, F_1384_=6.221, *P=*0.002). Overall, people with different academic education significantly differed in GAD-2 scores (Anova: F_4,1325_ =4.822, *P*<0.001), however post-hoc tests revealed considerable overlap between educational backgrounds, with a tendency for people with no education having higher GAD-2 scores (Tukeys B *P*<0.05). There was no effect for household size (ANOVA F_4_,_1398_=2.082, *P*= 0.081) or for the number of children (ANOVA F_3_,_1401_=0.545, *P*>0.05) on GAD-2 score.

In a multiple regression analysis, all predictors except for BMI were significant for GAD-2 score (r^2^ adjusted = 0.031, age, b=-0.019, t_1302_=-3.876, p<0.001; gender, b=0.083, t_1302_=2.929, p = 0.003; education, b=-0.84, t_1302_=-3.051, *P* = 0.002).
